# A Novel AP2/ERF Transcription Factor, OsRPH1, Negatively Regulates Plant Height in Rice

**DOI:** 10.3389/fpls.2020.00709

**Published:** 2020-05-27

**Authors:** Ziming Ma, Tao Wu, Kai Huang, Yong-Mei Jin, Zhao Li, Mojun Chen, Sokyong Yun, Hongjia Zhang, Xue Yang, Haoyuan Chen, Huijiao Bai, Lin Du, Shanshan Ju, Liping Guo, Mingdi Bian, Lanjuan Hu, Xinglin Du, Wenzhu Jiang

**Affiliations:** ^1^Jilin Province Engineering Laboratory of Plant Genetic Improvement, College of Plant Science, Jilin University, Changchun, China; ^2^Jilin Academy of Agricultural Sciences, Changchun, China; ^3^Kye Ung Sang College of Agriculture of Kim II Sung University, Pyongyang, North Korea

**Keywords:** AP2/ERF transcription factor, OsPRH1, plant height, OsCRY1b, rice

## Abstract

The APETALA 2/ethylene response factors (AP2/ERF) are widespread in the plant kingdom and play essential roles in regulating plant growth and development as well as defense responses. In this study, a novel rice AP2/ERF transcription factor gene, *OsRPH1*, was isolated and functionally characterized. OsRPH1 falls into group-IVa of the AP2/ERF family. OsRPH1 protein was found to be localized in the nucleus and possessed transcriptional activity. Overexpression of *OsRPH1* resulted in a decrease in plant height and length of internode and leaf sheath as well as other abnormal characters in rice. The length of the second leaf sheath of *OsRPH1*-overexpressing (OE) plants recovered to that of Kitaake (non-transgenic recipient) in response to exogenous gibberellin A_3_ (GA_3_) application. The expression of GA biosynthesis genes (*OsGA20ox1*–*OsGA20ox4, OsGA3ox1*, and *OsGA3ox2*) was significantly downregulated, whereas that of GA inactivation genes (*OsGA2ox7*, *OsGA2ox9*, and *OsGA2ox10*) was significantly upregulated in *OsRPH1-*OE plants. Endogenous bioactive GA contents significantly decreased in *OsRPH1-*OE plants. OsRPH1 interacted with a blue light receptor, OsCRY1b, in a blue light-dependent manner. Taken together, our results demonstrate that OsRPH1 negatively regulates plant height and bioactive GA content by controlling the expression of GA metabolism genes in rice. OsRPH1 is involved in blue light inhibition of leaf sheath elongation by interacting with OsCRY1b.

## Introduction

AP2/ERF family, a plant-specific transcription factor superfamily, is characterized by a highly conserved APETALA 2 (AP2) DNA-binding domain (Nakano et al., 2006; Rashid et al., 2012; Gu et al., 2017). AP2/ERF transcription factors are involved in plant stress responses and govern plant growth and development (Hu et al., 2008; Bai et al., 2016; Xiao et al., 2016). Phylogenetic analysis has identified a total of 170 *AP2/ERF* genes in the rice genome, which could be divided into 11 groups, including four major groups (AP2, ERF, DREB, and RAV), 10 subgroups, and 2 soloists (Nakano et al., 2006; Rashid et al., 2012; Gu et al., 2017). Several AP2/ERF members have been reported to influence plant growth and development via regulation of plant hormone synthesis and signaling such as gibberellins (GA), auxin, cytokinin, and abscisic acid (ABA) (Rashotte et al., 2006; Kitomi et al., 2011; Qi et al., 2011; Gu et al., 2017). *OsEATB*, a rice *AP2/ERF* gene, restricts internode elongation by downregulating the GA biosynthetic gene, *ENT-COPALYL DIPHOSPHATE SYNTHASE 2* (*Os2*) (Qi et al., 2011). Another *ERF* gene, *SUBMERGENCE1A* (*SUB1A*), confers submergence tolerance in rice by limiting ethylene-promoted GA responses during submergence wherein *Sub1A* enhances the accumulation of GA signaling repressors *SLENDER RICE 1* (*SLR1*) and *SLR1 LIKE-1* (*SLRL1*) (Xu et al., 2006; Fukao and Bailey-Serres, 2008). In addition, some AP2 domain-containing transcription factors play important roles in ABA and GA antagonism. Overexpression of *ABA INSENSITIVE 4* (*ABI4*) promotes ABA biosynthesis and represses GA biosynthesis by activating of *9-CIS-EPOXYCAROTENOID DIOXYGENASE 6* (*NCED6*) and *GIBBERELLIN 2-OXIDASE 7* (*GA2ox7*) in Arabidopsis (Shu et al., 2016). Overexpression of *OsAP2-39* leads to overall biomass reduction, including seed yield, and OsAP2-39 directly activates the ABA biosynthesis gene *OsNCED1* and the GA inactivating gene *ELONGATED UPPERMOST INTERNODE1*(*OsEUI1*) (Yaish et al., 2010).

In cereal crops, dwarfism is a vital agronomic trait conferring resistance to wind and water lodging, thus contributing to yield (Khush, 2001; Sasaki et al., 2002). GA is one of the most important hormones affecting plant height. More than 100 GA species have been identified, among which GA_1_, GA_3_, GA_4_, and GA_7_ have been confirmed to be endogenously active in flowering plants (Hedden and Thomas, 2012). GA_1_ and GA_4_ are the main bioactive GAs in rice; both are involved in regulating vegetative growth (Kobayashi et al., 1988; Magome et al., 2013). In addition, GA_3_ and GA_7_ have been identified in rice, but GA_3_ plays a minor role and is present at much lower concentrations than GA_1_ (Kobayashi et al., 1991; Hasegawa et al., 1995; Itoh et al., 2001; Hedden, 2002). Furthermore, multiple enzymes are involved in GA biosynthesis, in which GA 3-oxidases (GA3oxs) catalyze the final step of GA biosynthesis, and GA 20-oxidases (GA20oxs) are responsible for producing the substrates for OsGA3oxs (Hedden and Thomas, 2012). GA 2-oxidases (GA2oxs) are critical for the inactivation of GA especially during vegetative growth (Thomas et al., 1999). Two *GA3ox* genes, four *GA20ox* genes, and 10 *GA2ox* genes have been identified in the rice genome (Sakamoto et al., 2004; Lo et al., 2008). The rice dwarf mutant *d18* and the green-revolution variety, *semi-dwarf 1* (*sd1*), are resulted from mutation in *OsGA3ox2* and *GA20ox2* genes, respectively (Itoh et al., 2001; Monna et al., 2002; Sasaki et al., 2002; Spielmeyer et al., 2002). *OsGA2ox5-*overexpressing rice plants exhibit dominant dwarf and GA-deficient phenotypes compared to the wild-type (Shan et al., 2014). *GIBBERELLIN INSENSITIVE DWARF1* (*GID1*) encodes a soluble GA receptor that mediates GA signaling in rice; loss-of-function mutations in *GID1* cause severely dwarfed plant height (Ueguchi-Tanaka et al., 2005). The rice GIBBERELLIN INSENSITIVE DWARF2 (GID2) is an F-box subunit of Skp1-Cullin-F box protein (SCF) E3 ubiquitin ligase, facilitating SLR1 degradation by the 26S proteasome in the presence of GA (Sasaki et al., 2003; Itoh et al., 2005). The *gid2* mutant, caused by the loss of function mutations in *GID2*, stunts severely, and leaves are broader, dark green in color (Sasaki et al., 2003).

According to the response to exogenous GA application, GA-related dwarf mutants can be divided into two types, namely, GA-deficient and GA-insensitive mutants. The GA-insensitive mutants are defective in GA signaling, and exogenous GA application does not rescue the GA-insensitive phenotypes, and the contents of endogenous bioactive GAs are usually much higher than those of wild-type (Ueguchi-Tanaka et al., 2000, 2005; Sasaki et al., 2003). However, GA-deficient mutants are caused by mutations in the enzymes involved in GA biosynthesis or GA inactivation. Phenotypes caused by insufficient bioactive GA levels *in vivo* can be restored by exogenous GA treatment (Ashikari et al., 2002; Sakamoto et al., 2004).

Blue light receptor cryptochromes regulate multiple aspects of plant growth and development (Liu et al., 2011). Three cryptochrome genes (*OsCRY1a*, *OsCRY1b*, and *OsCRY2*) have been identified in rice (Hirose et al., 2006). OsCRY1s regulate blue-light inhibition of coleoptile and leaf elongation, while OsCRY2 is involved in the promotion of flowering time in rice (Hirose et al., 2006; Zhang et al., 2006). Interestingly, OsCRY1s have been found to be essential for robust induction of the *GA2ox* genes and act with phytochromes cooperatively but independently to reduce bioactive GA content in rice seedlings in the light (Hirose et al., 2012). Similarly, cryptochromes in Arabidopsis mediate the blue light-induced *GA2ox1* expression and blue light suppression of *GA20ox1* and *GA3ox1* expression (Zhao et al., 2007). However, how blue light and OsCRY1 regulate the expression of these GA metabolism-related genes remains unclear.

In the present study, we report the identification and characterization of a novel AP2/ERF family transcription factor, Reduced Plant Height (OsRPH1), in rice. Overexpression of *OsRPH1* causes a decrease in plant height and internode length as well as other abnormal traits. Our results indicate that OsRPH1 negatively regulates plant height by controlling GA metabolism-related genes and is involved in OsCRY1b-mediated blue light inhibition of leaf sheath elongation in rice.

## Materials and Methods

### Plant Materials and Generation of *OsRPH1*-OE Rice

The coding sequence (CDS) of *OsRPH1* was cloned from 2-week-old rice seedlings of Kitaake (*Oryza sativa* L. subsp. *Japonica*) and inserted into the *Pst*I and *Spe*I sites of the vector pCUbi1390 under the control of the maize ubiquitin (*Ubi*) promoter. The primer sequences are listed in Supplementary Table S1. The constructed vector *pUbi:OsRPH1* was introduced into Kitaake by *Agrobacterium tumefaciens* (EHA105)-mediated transformation. Fifteen T_0_ lines were obtained, and 11 of these were shown to be positive by PCR amplification using normal sequencing primers adjacent to multiple clone sites of the vector pCUbi1390. The T_3_ homozygous lines were selected by hygromycin resistance evaluation.

### Growth Conditions, Exogenous GA_3_ Treatment, and Phenotype Analysis

The Kitaake (non-transgenic recipient) and the *OsRPH1-*OE transgenic seedlings were grown hydroponically in a growth chamber for 14 days under a condition of 12 h light at 30°C/12 h dark at 24°C, following by transplanting the seedlings into the field at Jilin University in Changchun, China, during the rice-growing season. The phenotype and agronomic traits were measured at different growth stages. A total of 10 plants for each line were measured, and each plant was measured three times. Fully developed grains were measured for grain length, width, and weight after being air-dried. A total of 20 grains from individual plants of each line were measured; each grain was measured three times. Student’s *t*-test was used to determine the statistical significance.

The exogenous GA_3_ rescue assay was performed as previously described with a few modifications (Qi et al., 2011). The grains were sterilized and planted in Kimura nutrient solution in the growth chamber. The 10-day-old seedlings were treated with 10, 50, and 100 μM GA_3_. At least 10 seedlings of each individual line were measured, and each seedling was measured for three times. The length of the second leaf sheath was measured at 6 or 12 h intervals. Student’s *t*-test was used to determine the statistical significance.

For the assay of 2nd leaf sheath elongation under blue light, the seeds were placed on a 96-well plate and cultured in Kimura nutrient solution for 2 days in the dark. These 2-day-old dark-grown seeds were exposed to blue light, white light, or dark conditions at 28°C for 6 d (LED light source, 20 μmol⋅m^–2^⋅s^–1^). The length of the second leaf sheath was measured. At least 20 seedlings of each individual line were measured. Student’s *t*-test was used to determine the statistical significance.

### Protein Structural and Phylogenetic Analysis

The protein structure of OsRPH1 was analyzed using online motif scan tool^1^. The homologous proteins of OsRPH1 were searched using BLASTP in the National Center for Biotechnology Information (NCBI)^2^ taking the full-length amino acid sequence of OsRPH1 as a query. Multiple protein sequence alignment was performed with DNAMAN. The phylogenetic analysis was constructed by MEGA version 4.0 with the bootstrap method based on full amino acid sequences. Bootstrap values evaluated for 1,000 bootstrap trails are shown at each node.

### Subcellular Localization and Bimolecular Fluorescence Complementation (BiFC) Assays

The CDS of *OsRPH1* was inserted into the pAN580-GFP vector driven by the CaMV35S promoter to form the OsRPH1-GFP construct. A D53-RFP fusion protein was used as the nuclear marker (Zhou et al., 2013; Duan et al., 2019). The cDNA fragment encoding OsCRY1b was amplified and cloned into apXY105 vector carrying the N-terminal half of YFP (nYFP) under the control of a CaMV35S promoter to form the nYFP-OsCRY1b, and the cDNA encoding OsRPH1 was cloned into a pxy103 vector carrying the C-terminal half of YFP (cYFP) under the control of CaMV35S promoter to form the cYFP-OsRPH1. nYFP-OsCRY1b and cYFP-OsRPH1 were co-transformed into rice protoplasts. As negative controls, nYFP and cYFP-OsRPH1, nYFP-OsCRY1b and cYFP were co-transferred to rice protoplasts separately. Rice protoplast preparation and transformation were performed as previously described by Wang et al. (2016). For subcellular localization assay, samples were incubated in the dark for 16 h at 23°C, and then the fluorescent images were captured using a confocal laser microscope (Carl Zeiss, LSM780). For BiFC assay, samples were incubated in the dark for 12–14 h at 23°C, then transferred to blue light (15 μmol⋅m^–2^⋅s^–1^) for 30 min or kept in darkness. The BiFC fluorescence signals were analyzed by Zeiss Axio Observer A1 (Carl Zeiss, Jena, Germany).

### Transactivation Activity and Yeast Two-Hybrid Assays

The CDS of *OsRPH1* was inserted into the pBridge vector to form pBridge-*OsRPH1*. SbSTOP1 (Huang et al., 2018) and the empty pBridge vector were, respectively, used as the positive and negative controls. These resultant constructs and empty pGADT7 vectors were transformed into the yeast strain AH109 (Clontech). The positive transformants were verified on double-dropout media (SD/-Trp-Leu) and then dropped on quatuor dropout media (SD/-Trp-Leu-Ade-His), which were performed according to the manufacturer’s user manual (Clontech, Mountain View, CA, United States). For yeast two-hybrid assay, the cDNA fragment encoding *OsRPH1* was amplified and cloned into pBridge vector to form pBridge-*OsRPH1* using Gateway recombination system as the “bait.” A cDNA library prepared from rice seedlings was used to perform the yeast two-hybrid screening, and positive clones were identified by DNA sequencing. The coding region of the target OsCRY1b was inserted into the pGADT7 vector as the “prey.” The “bait” and “prey” constructs were co-transformed into the yeast strain AH109. The positive transformants were verified on double dropout media (SD/-Trp-Leu) and then dropped on quatuor dropout media (SD/-Trp-Leu-Ade-His). Transformed yeast was incubated at 28°C in darkness or under blue light. All protocols were performed according to the manufacturer’s instructions (Clontech Laboratories, Mountain View, CA, United States).

Rice cDNA library was constructed as follows: Total RNA of 2-week-old rice seedlings was extracted, and then first-strand and double-stranded cDNA (dscDNA) was synthesized one after another. The dscDNA was homogenized using a Trimmer-Direct cDNA Normalization Kit (Evrogen, Moscow, Russia) after quality assessment. The dscDNA was digested with *Sfi*I and then purified. *Sfi*I-digested dscDNA was inserted into the pGADT7-*Sfi*I vector and then transformed into *E. coli* host strain. The transformed product was cultured on LB medium (Amp 100 g/L), and six single colonies grown on the plate were randomly selected to perform PCR amplification with pGADT7 primers: forward primer, 5′-GGAGTACCCATACGACGTACC-3′; reverse primer, 5′-TATCTACGATTCATCTGCAGC-3′).

### Quantification of Endogenous GAs

The levels of endogenous GAs were determined by high-performance liquid chromatography-tandem mass spectrometry (HPLC–MS/MS) according to the method described by Liu J. et al., 2018, with minor modifications. About 1 g of shoots harvested from 10-day-old rice seedlings was frozen and grounded to fine powder in liquid nitrogen. The tissue was extracted with the extraction solvent (isopropanol/hydrochloric acid, dichloromethane) and partitioned with centrifugation, and then the organic phase was retained. Under avoiding light conditions, organic phase was dried with nitrogen, dissolved in 400 μL of methanol (0.1% formic acid), and then purified with 0.22 μm filter membrane. The purified products were subjected to HPLC-MS/MS analysis using a poroshell 120 SB-C18 (Agilent Technologies, Palo Alto, United States) column (2.1 mm × 150 mm; 2.7 μm). The mobile phase was composed of solvent A (methanol and 0.1% methanoic acid) and solvent B (ultrapure water and 0.1% methanoic acid). MS analysis was performed using SCIEX QTRAP 6500. Three biological replicates and three technical replicates of each sample were used, and Student’s *t*-test was employed to determine the statistical significance of differences.

### Quantitative Real-Time PCR (qRT-PCR) Analysis

Total RNA was isolated from 14-day-old seedlings of Kitaake and *OsRPH1-*OE lines using an RNA Prep Pure Plant Kit (Tiangen Co., Beijing, China) and was reverse transcribed using a SuperScript II Kit (TaKaRa, Tokyo, Japan). qRT-PCR was performed using an SGExcel Fast SYBR qPCR Mixture (Sangon Biotech, Shanghai, China) on an Agilent Strata Gene Mx3005PMx3000P (USA). Each reaction contained 10 μL of SGExcel Fast SYBR qPCR Mixture, 0.2 μM of each primer, and 1 μL template cDNA. The rice ubiquitin gene (*Os03g0234200*) was used as internal control (Zheng et al., 2015). All experiments were conducted using two biological replicates and four technical replicates for each sample. The relative quantification method (ΔΔC_T_) was applied to evaluate the quantitative variation among replicates. The primers used in the qRT-PCR are listed in Supplementary Table S1.

## Results

### Overexpression of *OsRPH1* Reduced Plant Height of Rice

**FIGURE 1:**
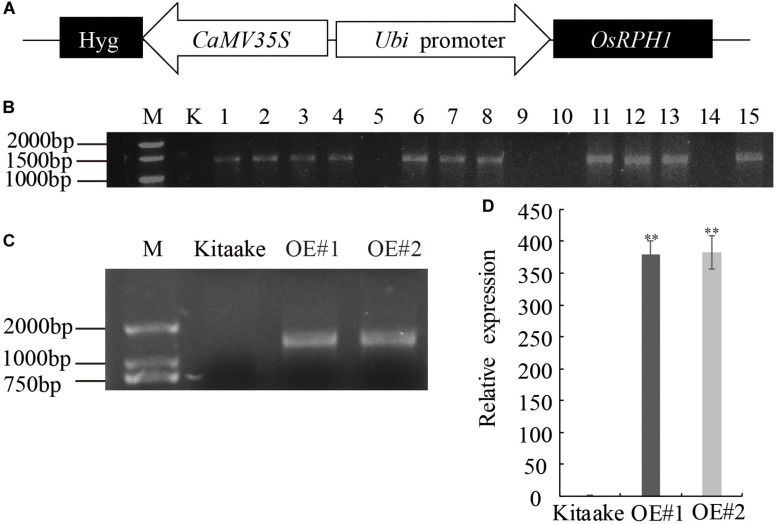
Generation of *OsRPH1-*OE rice. **(A)** Schematic representation of the *pUbi:OsRPH1* plant expression vector. The *OsRPH1* under the control of maize ubiquitin (Ubi) promoter and the hygromycin (hyg) gene under the control of 35S promoter were indicated. **(B)** PCR analyses for T_0_ transgenic rice using construct-specific primers. M: marker; K, Kitaake (non-transgenic recipient); 1–15: T_0_ generation of independent transgenic rice plants. **(C)** The *OsRPH1-*OE lines of OE#1 and OE#2 were confirmed by PCR using genomic DNA as the template. M: marker; Kitaake (non-transgenic recipient). **(D)** The expression of *OsRPH1* was quantified by qRT-PCR using 14-day-old rice seedlings. The data are presented as the mean ± SE values (*n* = 3) of two independent experiments. Double asterisks indicate significant differences at *P* ≤ 0.01 compared with Kitaake in Student’s *t*-test.

In our recent research, a number of predicted transcription factor genes have been isolated, and their overexpressing transgenic rice libraries were constructed by *Agrobacterium-*mediated transformation. Among these, a novel expressed gene of *LOC_Os05g49700* was transformed into Japonica rice variety Kitaake under the control of maize ubiquitin promoter (Figure 1A). Fifteen independent T_0_ lines of *LOC_Os05g49700-*OE pants were obtained, and 11 of them were proved to be positive by PCR amplification with specific primers adjacent to multiple clone sites of plasmid (Figure 1B). The homozygous lines of transgenic plants, which were selected by hygromycin resistance evaluation, exhibited a significant decrease in plant height; therefore, *LOC_Os05g49700* was designated as *REDUCED PLANT HEIGHT1*(*OsRPH1*). Two independent *OsRPH1-*OE lines (OE#1 and OE#2) with high mRNA expression levels were selected for further analysis (Figures 1C,D).

**FIGURE 2:**
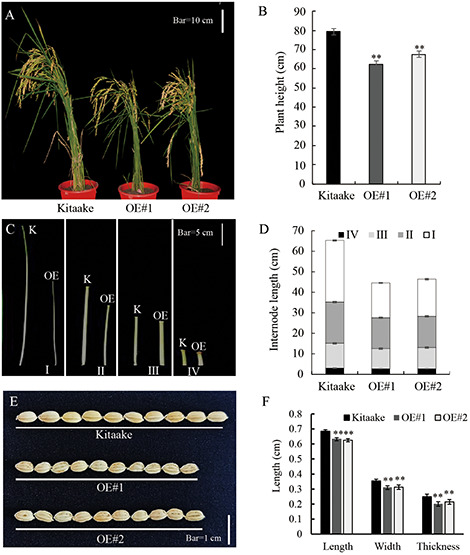
Morphology of *OsRPH1-*OE lines and Kitaake. **(A,B)** Whole-plant heights of *OsRPH1-*OE lines and Kitaake at maturity. A total of 10 plants for each line were measured; each plant was measured thrice. Mean values were calculated from the measurement on 10 plants. Bar = 10 cm. **(C,D)** Internodes length of main culms of Kitaake (K) and OE#1 (OE). A total of 10 plants for each line were measured; each plant was measured thrice. Bar = 5 cm. Mean values were calculated from the measurement of 10 plants. **(E,F)** Grain size of *OsRPH1-*OE lines and Kitaake. A total of 20 grains from individual plants for each line were measured, each grain was measured thrice. Bar = 1 cm. Double asterisks indicate significant differences at *P* ≤ 0.01 compared with Kitaake in Student’s *t-*test.

Compared to Kitaake, the *OsRPH1*-OE lines exhibited reduced plant height at all growth stages. At maturity, the average plant height of OE#1 (62.3 cm) and OE#2 (67.5 cm) was only 78.6 and 85.1% of Kitaake (79.3cm), respectively (Figures 2A,B). A comparison of internode length showed that the three internodes (first, second, and third internodes) of the stems in the *OsRPH1-*OE lines were significantly shorter than Kitaake (*P* ≤ 0.01); for instance, the average length of the second stem internodes of Kitaake was 20.1 cm, whereas the counterparts of two *OsRPH1-*OE lines were 15.0 and 15.2 cm (Figures 2C,D). *OsRPH1-*OE lines showed significantly shorter grain length, width, and thickness than Kitaake (*P* ≤ 0.01) (Figures 2E,F). Compared with Kitaake, OE#1 and OE#2 presented inhibition of the second leaf sheath elongation at the early growth stage (Figures 9A,D). These results indicate that overexpression of *OsRPH1* disrupts plant height and other agronomy traits in rice.

### *OsRPH1* Encodes an AP2/ERF Transcription Factor

**FIGURE 3:**
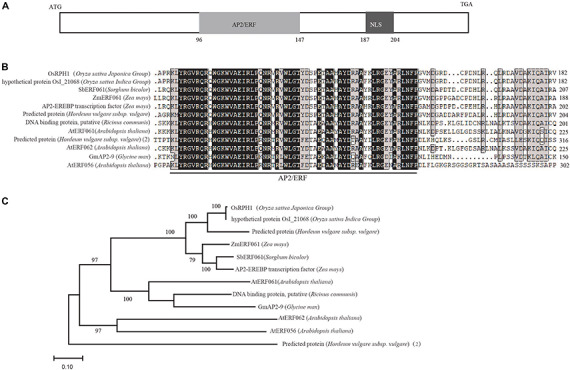
OsRPH1 encodes an AP2ERF protein. **(A)** The predicted domains of OsRPH1. NLS, Nuclear localization signal. The numbers indicate the amino acid positions of these domains. **(B)** Multiple sequence alignment of the AP2/ERF region of OsRPH1 with other AP2/ERF proteins in plants. The AP2/ERF domain is indicated, and identical and conserved amino acids are displayed in black and gray backgrounds, respectively. **(C)** The phylogenetic tree of OsRPH1 and its orthologous proteins in plants. The phylogenetic analysis was constructed using MEGA version 4.0 with the bootstrap method based on full amino acid sequences. Bootstrap values evaluated for 1,000 bootstrap trails are shown at each node. The following protein sequences are incorporated into the analysis: rice OsI_21068 (*Oryza sativa Indica Group*, gb| EAY99109), Sorghum SbERF061 (*Sorghum bicolor*, gi| 8074620), maize ZmERF061 (*Zea mays*, gi| 103635354), maize AP2-EREBP transcription factor (*Zea mays*, gi| 100272581), barley predicted protein (*Hordeum vulgare* subsp. *Vulgare*, gb| BAK02927), castor DNA binding protein, putative (*Ricinus communis*, gi| 8287762), Arabidopsis AtERF061 (*Arabidopsis thaliana*, gi| 842745), Arabidopsis AtERF062 (*Arabidopsis thaliana*, gi| 826996), soybean GmAP2-9 (*Glycine max*, gb| ACJ37443), Arabidopsis AtERF056 (*Arabidopsis thaliana*, gi| 816754), barley predicted protein(2) (*Hordeum vulgare* subsp. *vulgare*) BAJ98638.

A search of Rice Expression Profile Database^3^ showed that *OsRPH1* was expressed in various organs throughout plant development, with the higher expression in leaves and leaf sheaths (Supplementary Figure S1). *OsRPH1* has no introns, and the full-length CDS of *OsRPH1* is 822 nucleotides. It encodes a protein of 273 amino acids, with a calculated molecular mass of 29.71 kD and contained one AP2/ERF domain and one putative nuclear localization signal (NLS)^4^ (Figure 3A). Multiple protein sequences alignment showed that AP2/ERF domain of OsRPH1 had high amino acid similarity with its orthologous genes in sorghum, corn, soybean, and other plants (Figure 3B). OsRPH1 had been identified as OsAP2/EREBP-53, which fell into group-IVa of AP2/ERF based on domain similarities (Rashid et al., 2012). This group consists of 10 genes, and OsRPH1 has low homology with other genes in this group (Rashid et al., 2012). Phylogenetic analysis based on the alignment of full amino acid sequences revealed that OsRPH1 was most similar (69.02%) with a predicted AP2/ERF protein with unknown function in barley (Figure 3C).

### OsRPH1 Functions as a Transcription Activator

**FIGURE 4:**
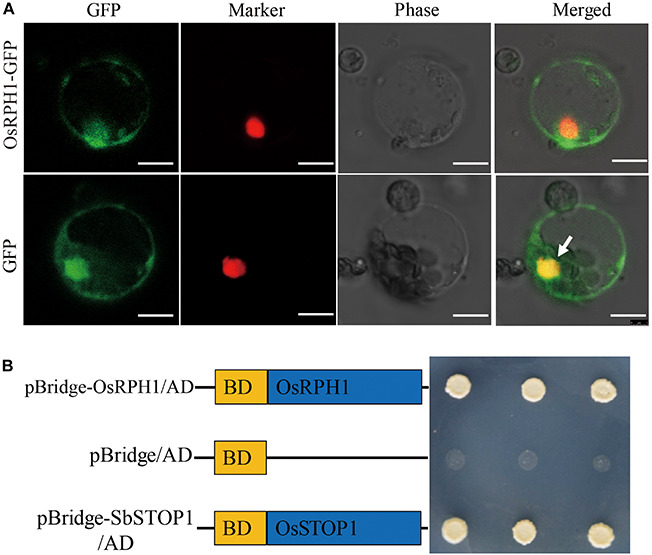
Subcellular localization and transcriptional activity of OsRPH1. **(A)** Subcellular localization of OsRPH1-GFP fusion proteins in rice protoplasts. D53-RFP was used as nuclear marker, Bar = 10 μm. **(B)** Transcriptional activity of OsRPH1 protein in yeast. BD, GAL4-DNA binding domain. The SbSTOP1 protein was used as a positive control.

It is well-known that transcription factors regulate gene expression by binding to *cis*-elements on genomic DNA sequences in the nucleus. To verify the subcellular localization of the OsRPH1 protein, the coding sequence of *OsRPH1* fused with GFP in the C-terminus under the control of the CAMV35S promoter (*p35S::OsRPH1-GFP*) was generated, and then transiently expressed in rice protoplasts. The results showed that the OsRPH1-GFP fusion protein is mainly located in the nucleus of rice protoplasts, indicating that OsRPH1 is a nuclear-localized protein (Figure 4A).

To determine whether the OsRPH1 protein has transactivation activity, OsRPH1 was fused with the GAL4-DNA binding domain and then expressed in yeast. Figure 4B shows that the transformants of pBridge-OsRPH1 and the positive control grew well on quatuor dropout media (SD/-Trp-Leu-Ade-His). However, the negative control failed to grow (Figure 4B). These results strongly support the hypothesis that OsRPH1 functions as a transcriptional activator in the nucleus.

### The Dwarf Phenotype of *OsRPH1-*OE Plants Is Rescued by the Application of Exogenous GA_3_

**FIGURE 5:**
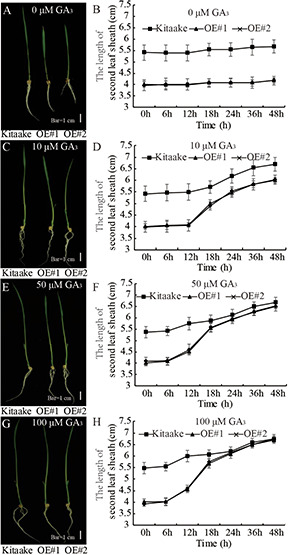
The exogenous GA_3_ rescue assay. The 10-day-old seedling of OE lines and Kitaake was treated with 0 **(A,B)**, 10 **(C,D)**, 50 **(E,F)**, and 100 μM **(G,H)** GA_3_. At least 10 rice seedlings of each individual line were measured, and each seedling was measured thrice in each experiment. Bar = 1 cm. Data are presented the average of 10 samples per genotype (±SD).

GA-deficient plants are dwarfed (Spielmeyer et al., 2002). To examine the sensitivity of *OsRPH1*-OE plants to GA, the second leaf sheath elongation induced by exogenous GA_3_ application was examined. Ten-day-old seedlings of *OsRPH1*-OE lines and Kitaake were treated with different concentrations of GA_3_ (0, 10, 50, and 100 μM), and the length of the second leaf sheath was investigated. The second leaf sheath length of *OsRPH1*-OE lines rapidly elongated within 24 h of GA_3_ treatment. Interestingly, under the concentration of 100 μM, the lengths recovered to that of the Kitaake (Figure 5). These results showed that the application of exogenous GA_3_ could completely restore the shortened second leaf sheaths of *OsRPH1*-OE plants, indicating that the dwarf phenotype of *OsRPH1*-OE plants might be caused by endogenous GA deficiency, not by repression of GA signaling.

### Expression of GA Metabolism-Related Genes Are Regulated by *OsRPH1* Overexpression

**FIGURE 6:**
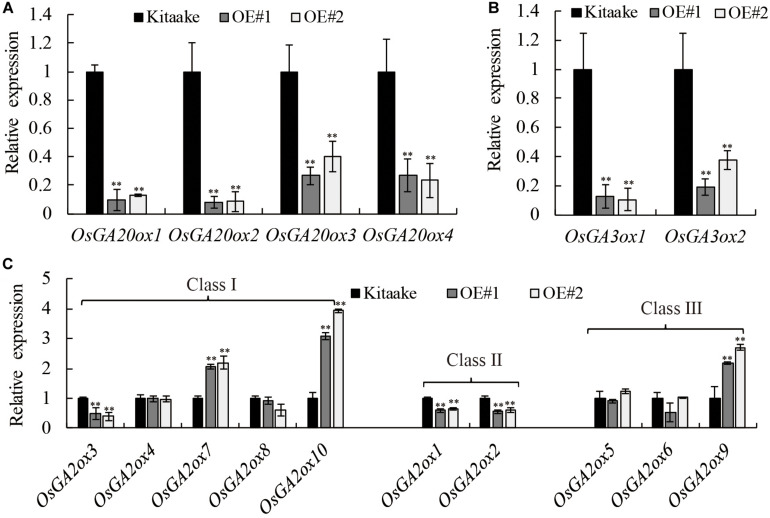
Expression analyses of the genes involved in **(A,B)** GA synthesis genes and **(C)** GA inactivation genes. All experiments were conducted in two biological replicates and four technical replicates for each sample. The relative quantification method (ΔΔC_T_) was applied to evaluate the quantitative variation among replicates. Transcript levels from Kitaake were set to 1. Data are expressed as mean ± SD (*n* = 3). Student’s *t*-tests were used to generate the *P*-values. ^∗∗^ Indicate significant differences between Kitaake and overexpressing-*OsRPH1* plants at *P* ≤ 0.01.

Many GA responsive dwarf plants that are deficient in the biosynthesis of active GAs have been characterized in various plant species (Ross et al., 1997; Qin et al., 2013). To determine whether GA metabolism was affected in *OsRPH1*-OE plant, transcript level of GA metabolism-related genes including four *OsGA20ox* members, two *OsGA3ox* members, and 10 *OsGA2ox* members was analyzed using qRT-PCR in 10-day-oldseedlings. Figure 6 shows that the expression level of *OsGA20ox1*–*OsGA20ox4* and *OsGA3ox1* and *OsGA3ox2* was all significantly (*P* ≤ 0.01) downregulated in *OsRPH1*-OE lines compared to Kitaake. Among the members of *OsGA20ox* family, the expression level of *OsGA20ox1* and *OsGA20ox2* was more strongly downregulated than *OsGA20ox3* and *OsGA20ox4* in *OsRPH1-*OE lines (Figure 6A).

Concomitantly, the expression level of GA inactivation genes, *OsGA2ox7*, *OsGA2ox9*, and *OsGA2ox10*, was significantly (*P* ≤ 0.01) upregulated in *OsRPH1*-OE lines, reaching about 2.1–3.5-fold of those in Kitaake. In contrast, the expression level of *OsGA2ox1–OsGA2ox3* was not clear. The transcripts of the remaining members, *OsGA2ox4–OsGA2ox6* and *OsGA2ox8*, could be observed, while was not significantly different between *OsRPH1*-OE lines and Kitaake (Figure 6C). These results suggested that *OsRPH1* negatively regulates the expression of four GA biosynthesis genes and positively regulates three GA inactivation genes in rice.

### Contents of Bioactive GAs Were Reduced in *OsRPH1-*OE Plants

**FIGURE 7:**
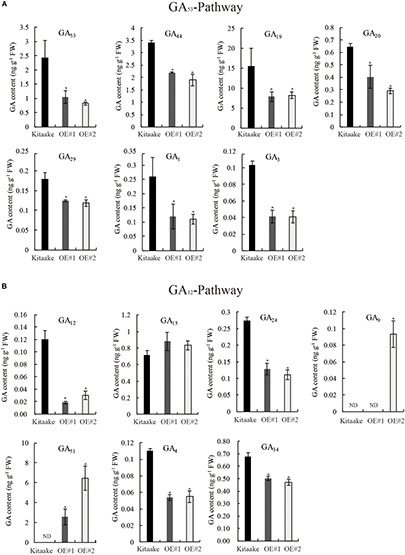
Quantification of endogenous GA in seedling stage of *OsRPH1*-OE lines and Kitaake. **(A)** Contents of GA species in GA_53_-pathway. **(B)** Contents of GA species in the GA_12_ pathway. Three biological replicates and three technical replicates on each sample were performed, and Student’s *t*-tests were used to determine statistical significance. ND, not detected; FW, fresh weight; error bars indicate ± SE (*n* = 3). Single asterisk indicates significant differences at *P* ≤ 0.05 compared with Kitaake in Student’s *t-*test.

To identify alterations in bioactive GA levels caused by *OsRPH1* overexpression, endogenous GA content in 10-day-old seedlings of rice was determined by HPLC-MS/MS. The GA species belonging to early-13-hydroxylation (GA_53_-) and non-13-hydroxylation (GA_12_-) pathways were detected, and the concentration of bioactive GAs and intermediates is shown in Figure 7. The level of bioactive GA_1_, GA_3_ and their precursors (GA_20_, GA_19_, GA_44_, and GA_53_ in the GA_53_ pathway) (Figure 7A), and bioactive GA_4_ and its precursors (GA_9_, GA_24_, GA_12_, in the GA_12_ pathway) (Figure 7B) decreased in *OsRPH1*-OE lines compared to Kitaake (Figure 7), which coincided with the lower level of *GA20ox* and *GA3ox* genes (Figure 6). The expression of GA 2-oxidase product GA_51_ significantly increased (Figure 7B), which is concordant with the higher level of *GA2ox* (Figure 6C). However, GA_29_ and GA_34_ showed a significant decreased in content (*P* ≤ 0.05) in *OsRPH1-*OE lines, which may be related to their low substrate level (Figure 7). GA_8_ was not detected in all three samples. These results indicate that the overexpression of *OsRPH1* reduces the level of bioactive GAs (GA_1_, GA_3_, and GA_4_) by downregulating GA biosynthetic genes and upregulating GA inactivation genes in the transgenic rice, which in turn results in reduced plant height.

### OsRPH1 Interacts With Blue Light Receptor OsCRY1b

**FIGURE 8:**
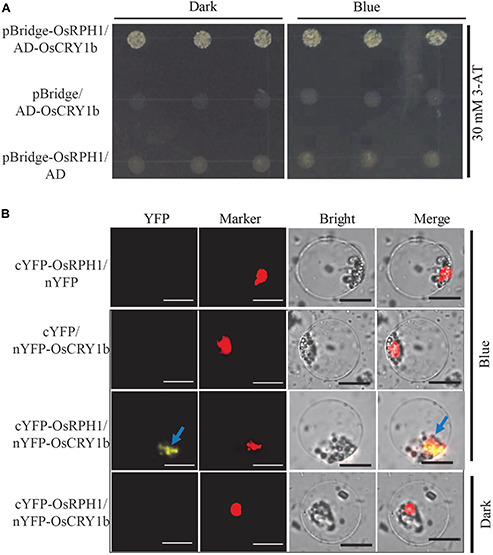
Protein interaction between OsRPH1 and OsCRY1b in yeast and plant cells. **(A)** The interaction between OsRPH1 and OsCRY1b was detected in yeast cells. Yeast two-hybrid analyses of the indicated proteins on minimal synthetic defined media containing -Trp-Leu or -Trp-Leu-Ade-His dropout supplements with 30 mM 3-AT. AD, activation domain; BD, GAL4-DNA binding domain. **(B)** BiFC assays showing the association of OsRPH1 and OsCRY1b in rice protoplasts under blue light and dark conditions. YFP, YFP fluorescence; Marker, marker fluorescence; Bright, bright field. Bar = 10 μm.

To identify the interactive protein of OsRPH1, the GAL4 yeast two-hybrid system was used to screen for the candidates in rice cDNA library, using full-length OsRPH1 as bait. A total of three targets were detected, including OsCRY1b and two unknown proteins with the AdoMetDC_leader and SAM_decarbox domains (PFAM) and DUF1084 domain (PFAM), respectively (Supplementary Table S2). OsCRY1b is a blue light receptor that is vital in regulating rice growth and development (Hirose et al., 2006; Zhang et al., 2006). Therefore, OsCRY1b was selected for further investigation. The coding sequence of OsCRY1b was cloned into the prey vector, and the yeast two-hybrid assay was performed. Figure 8A shows that by adding 3-amino-1, 2, 4-triazole (3-AT) to the medium effectively inhibited the self-activation of OsRPH1; the interaction between OsRPH1 and OsCRY1b was observed in yeast cells. OsRPH1-OsCRY1b interaction occurs under both darkness and blue light conditions in yeast cells (Figure 8A). To further identify whether the interaction of OsRPH1 with OsCRY1b happens *in vivo*, the BiFC assay was performed in rice protoplasts under darkness and blue light conditions. Interestingly, the fluorescence signal of YFP was evidently detected under blue light in the nucleus of protoplast, whereas no signal was detected under darkness (Figure 8B). These results suggest that OsRPH1 interacts with OsCRY1b in rice protoplasts in a blue light-dependent manner.

### Overexpression of *OsRPH1* Increases Sensitivity to Blue Light

**FIGURE 9:**
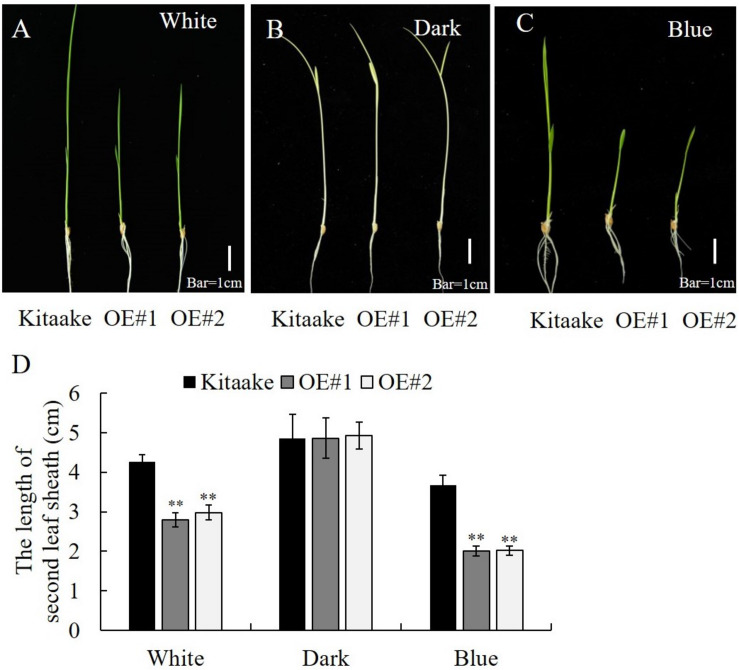
Length of leaf sheath in *OsRPH1-*OE lines and Kitaake that were grown for 6 days under white light **(A)**, dark **(B)**, or blue light **(C)**. Bar = 1 cm. **(D)** The second leaf sheath length of rice seedlings under white light **(A)**, dark **(B)**, or blue light **(C)** for 6 days was measured. Data are mean (±SD) of 10 samples. ^∗∗^ Indicates that the significant differences between *OsRPH1-*OE plants and Kitaake.

OsCRY1b mediates blue light-dependent inhibition of coleoptile and leaf elongation in rice (Hirose et al., 2006). Here, we tested whether *OsRPH1*-OE lines exhibit a growth inhibition phenotype in a blue light-dependent manner. The seedlings of *OsRPH1*-OE lines and Kitaake were grown under white light, blue light, or darkness conditions, and the length of the second leaf sheath was measured after 6 days. There was no significant difference of the second leaf sheath length between *OsRPH1*-OE lines and Kitaake under the darkness condition (Figure 9B). In contrast, under white light and blue light conditions, *OsRPH1*-OE lines had shorter leaf sheaths than Kitaake (Figures 9A,C). As expected, this phenotype was much more pronounced under blue light conditions than under white light condition, which demonstrated that the average length of the second leaf sheath of OE#1 (2.75 cm) and OE#2 (2.97 cm) was 64.7 and 69.9% of the Kitaake (4.25 cm) under white light condition, whereas the counterparts under blue light were 55.8 and 55.3% (Figure 9D). These results indicate that overexpression of *OsRPH1* accelerates the inhibition of the second leaf sheath elongation under blue light, suggesting that OsRPH1 is associated with blue light-inhibited cell elongation through interacting with OsCRY1b.

## Discussion

AP2/ERF transcription factor family regulates agronomic traits, including plant growth, defense responses, and fruit ripening through mediating phytohormonal biosynthesis and signals (Gu et al., 2017). A total of 170 *AP2/ERF* genes have been identified in rice genome, and several AP2/ERF members play roles in controlling plant growth and development via integrating different phytohormonal signals (Xu et al., 2006; Fukao and Bailey-Serres, 2008; Yaish et al., 2010; Qi et al., 2011). miR172-mediated HvAP2 controls internode elongation in barley (Patil et al., 2019). However, most of the rice AP2/ERF members have not been studied to date. Here, we provide evidence that OsRPH1, which belongs to group-IVa of the AP2/ERF family, functions as a transcription factor that regulates plant height at least by reducing bioactive GA contents. In addition, our results suggest that OsRPH1 is closely related to blue light signal-triggered inhibition of leaf sheath elongation in rice seedlings, which is mediated by the blue light receptor OsCRY1b.

GA is a predominant growth-promoting phytohormone that regulates leaf sheath and internode elongation in rice. Rice mutants defective in GA biosynthesis or perception exhibit dwarf phenotypes (Hedden, 2003; Ueguchi-Tanaka et al., 2005; Liu C. et al., 2018); among them the dwarfing mutants related to GA synthesis can be restored to their wild-type by exogenous GA application, whereas GA-insensitive mutants do not (Gomi et al., 2004). *OsRPH1*-OE rice exhibited plant height reduction, internode length shortening, and other abnormal characters (Figure 2), as well as shortened leaf sheath (Figure 9). Moreover, exogenous application of GA_3_ could completely restore the short second leaf sheath of *OsRPH1*-OE plants to that of the Kitaake (Figure 5), which implies that OsRPH1 may be involved in GA metabolism instead of GA signaling.

The regulatory mechanism of OsRPH1 on GA metabolism gene expression is both positive and negative. GA biosynthesis genes were downregulated (Figures 6A,B) whereas GA inactivation genes were upregulated in the *OsRPH1*-OE lines compared to Kitaake (Figure 6C). Mutations in GA biosynthesis genes *GA20ox* and *GA3ox* in maize (*dwarf1*) and barley (*sdw1/denso*), respectively, could reduce plant height (Spray et al., 1996; Jia et al., 2015). Additionally, overexpression of *OsGA2ox1*, *OsGA2ox6*, or *OsGA2ox9* genes in rice also causes moderate height reductions (Sakamoto et al., 2001; Sakai et al., 2003; Lo et al., 2008; Huang et al., 2010). These studies suggest that GA biosynthesis gene mutation and GA inactivation gene overexpression can reduce plant height. These two circumstances simultaneously occur in *OsRPH1*-OE plants.

The differential expression of GA oxidase genes in *OsRPH1*-OE plants relative to Kitaake (Figure 6) may lead to a decrease in bioactive GA content (Figure 7), which is closely related to lower plant height (Figure 2). In addition, the level of deactivated product GA_51_ significantly increased (Figure 7B), which coincides with the upregulation of the *GA2ox* genes (Figure 6C). However, compared with Kitaake, the contents of GA intermediates, GA_53_ and GA_12_, decreased in the *OsRPH1*-OE lines, but there was no accumulation, which is discordant with the downregulation of *GA20ox* genes. There are two possible reasons for this biological phenomenon: one is that the enzymes in the early steps of GA biosynthesis may also be affected, and the other is that GA_53_ and GA_12_ may be converted into GA_97_ and GA_110_, respectively, by high *GA2ox* activities. *Rht18* semi-dwarfism in wheat is due to an increase in *GA2oxA9*, which converts the intermediate GA_12_ to the inactive metabolite GA_110_ (Ford et al., 2018). *SoGA2ox3* from spinach could hydroxylate GA_12_ and GA_53_, and its overexpression in *Nicotiana sylvestris* produces high level of GA_97_ with a concomitant decrease in active GA_1_ level that result in GA-deficient phenotypes (Lee and Zeevaart, 2005).

In this study, a blue light receptor, OsCRY1b, was found to interact with OsRPH1 in both yeast cells and rice protoplasts. However, the blue light-dependent interaction between OsRPH1 and OsCRY1b was only observed in rice protoplasts and not in yeast cells (Figure 8). The reason of this is probably due to their differential expression systems. OsCRY1b mediates blue light perception to inhibit the elongation of coleoptiles, leaf sheaths, and blades by inducing the expression of *OsGA2ox4*–*OsGA2ox7* genes (Hirose et al., 2006, 2012; Zhang et al., 2006). Cryptochrome signals induce several *GA2ox* genes to decrease bioactive GA level through two processes, including the reduction of precursor GA species by class III enzymes and bioactive GA species directly by class I enzymes (Hirose et al., 2012). Similarly, the expression of *GA2ox* genes, *OsGA2ox7* and *OsGA2ox10* (class I) and *OsGA2ox9* (class III), was significantly upregulated in *OsRPH1*-OE lines than Kitaake (Figure 6B). In addition, cryptochromes mediate the blue light-induced *GA2ox1* and blue light suppression of *GA20ox1* and *GA3ox1* in Arabidopsis (Zhao et al., 2007). Similarly, the expression of all the *GA20ox* and *GA3ox* genes was significantly downregulated in *OsRPH1*-OE lines compared with Kitaake (Figure 6A). Furthermore, the inhibition of leaf sheath elongation caused by the overexpression of *OsRPH1* under blue light condition was stronger than that under white light condition (Figures 9A,C,D). These results imply that OsRPH1 is implicated in blue light signal transduction in rice. The interaction between OsRPH1 and OsCRY1b promotes the decrease in bioactive GA content by regulating GA biosynthesis and GA inactivation gene expression, which leads to decreased plant height and leaf sheath length. AP2/ERF proteins can bind a *cis*-regulatory element, GCC-box (AGCCGCC), which was originally identified as an ethylene response element (Gu et al., 2017). In rice, OsAP2-39 strongly binds to the GCC box motif, which is present in the promoters of its target genes, *OsNCED-1* and GA inactivation gene *OsEUI1* (Yaish et al., 2010). Also, OsEATB specifically binds to the GCC box (Qi et al., 2011). All four genes of *OsGA20ox* and both of *OsGA3ox* exhibited reduced transcript abundance in OE lines relative to Kitaake (Figure 6), and all these six genes contain at least one GCC box element in their promoter regions (data not shown). This means that OsRPH1 may have a common regulatory pattern between *GA20ox* and *GA3ox* genes and may directly or indirectly regulate the expression of these genes in combination with GCC box, but its real regulatory mechanism remains unclear.

In summary, OsRPH1 is a novel AP2/ERF transcription factor that negatively regulates plant height by modulating bioactive GA contents. OsRPH1 downregulates *OsGA20ox* and *OsGA3ox* genes, and upregulates *OsGA2ox* genes. The mechanism of OsRPH1-regulated GA metabolism and the effect of OsRPH1-OsCRY1b interaction on plant growth and development should be clarified in the next study.

## Data Availability Statement

All datasets generated for this study are included in the article/Supplementary Material.

## Author Contributions

ZM, TW, Y-MJ, XD, and WJ participated in the experimental design. ZM, KH, Y-MJ, ZL, MC, SY, HZ, XY, HC, HB, LD, SJ, and LG performed the research. ZM, TW, Y-MJ, MB, LH, XD, and WJ participated in the manuscript writing and amending.

## Conflict of Interest

The authors declare that the research was conducted in the absence of any commercial or financial relationships that could be construed as a potential conflict of interest.

## Footnotes

1 https://myhits.isb-sib.ch/cgi-bin/PFSCAN

2 https://blast.ncbi.nlm.nih.gov/Blast.cgi

3 http://ricexpro.dna.affrc.go.jp/

4 http://www.ricedata.cn/gene/gene_info.aspx?id=LOC_Os05g49700

## Supplementary Material

FIGURE S1 Expression of *OsRPH1* in various tissues.

TABLE S1 Sequences of the primers used in this study.

TABLE S2 Potential proteins interacted with OsRPH1.
