# Supplementary figures and images for: A Novel AP2/ERF Transcription Factor, OsRPH1, Negatively Regulates Plant Height in Rice
Source: Front Plant Sci. 2020 May 27;11:709. doi: 10.3389/fpls.2020.00709 (PMC7266880; doi:10.3389/fpls.2020.00709)

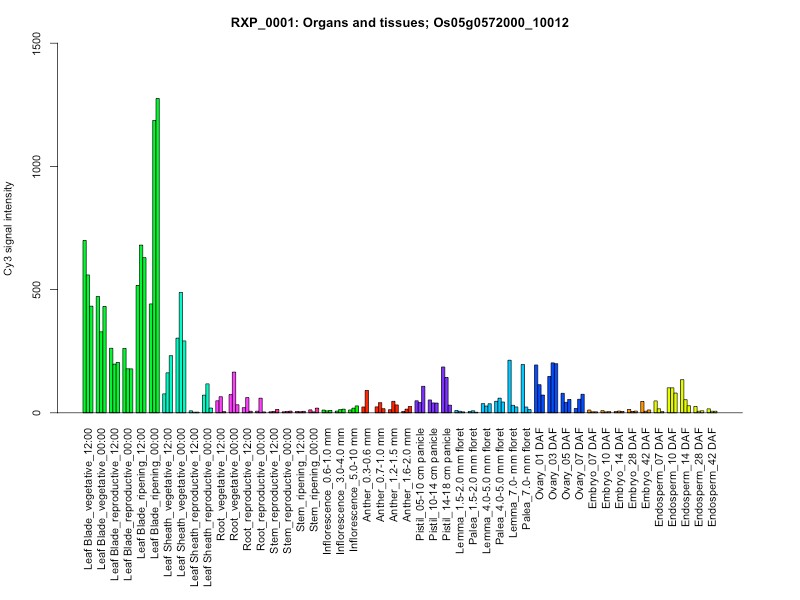

Supplement: FIGURE S1 — Expression of OsRPH1 in various tissues. [file Image_1.JPEG]
