# Supplementary material for: A Novel AP2/ERF Transcription Factor, OsRPH1, Negatively Regulates Plant Height in Rice
Source: Front Plant Sci. 2020 May 27;11:709. doi: 10.3389/fpls.2020.00709 (PMC7266880; doi:10.3389/fpls.2020.00709)
Supplement: TABLE S1 — Sequences of the primers used in this study. [file Table_1.DOCX]

**Supplementary TABLE 1 | Sequences of the primers used in this study**

| Experiments | Primer names | Primer sequences (5’-3’) |
| --- | --- | --- |
| The construct specific primer | 1390-F | TGCCTTCATACGCTATTTATTTGC |
|  | 1390-R | GTTAACACTAGTCAGATCTACCATG |
| Plant Expression Vector Construction | PUbi:OsRPH1-F | GGGGTACCATGGACGCCAGCCTCC |
|  | PUbi:OsRPH1-R | GGACTAGTGAAGTTAAGCATCTCCCA |
| Real-time PCR | OsGA20ox1F | CACTACAGGGCCGACATG |
|  | OsGA20ox1R | TATATTGTTGGTTGCAGGTGAC |
|  | OsGA20ox2F | GACTACTTCTCCAGCACC |
|  | OsGA20ox2R | CTTCATCTCCTCGCAGTA |
|  | OsGA20ox3F | GTCGGACACCAACACCAT |
|  | OsGA20ox3R | GTACTTGTCGAGGCTTTCATAG |
|  | OsGA20ox4F | GATTACTTCTCCACCCTAG |
|  | OsGA20ox4R | CATTATCGCCTTCGTCAC |
|  | OsGA3ox1F | GCTGTAAGGGATAAGTTGTT |
|  | OsGA3ox1R | ACTCTCCTTGTCCTCTTC |
|  | OsGA3ox2F | ACGACTACCTCCTCTTCT |
|  | OsGA3ox2R | GAACAACCTCAGCAACTC |
|  | OsGA2ox1F | CTCGCTTCAGTGCTATTG |
|  | OsGA2ox1R | CTAACAGGTCCAGGATCT |
|  | OsGA2ox2F | GTTCAGCCAGGTGGTAAA |
|  | OsGA2ox2R | CTCTCCTAGCAGGTCAAG |
|  | OsGA2ox3F | CAATGGGAGGTTCAAGAG |
|  | OsGA2ox3R | CCAAAGTAGATGAAGGAAAC |
|  | OsGA2ox4F | GAACGGGAGGATGAGGAG |
|  | OsGA2ox4R | TCCGAAGTAGATCATTGACAC |
|  | OsGA2ox5F | CTACCACACGCTCATCATC |
|  | OsGA2ox5R | TACTCGCCGAAGGTGAAG |
|  | OsGA2ox6F | AAGAAGGTGCAGGAAGAC |
|  | OsGA2ox6R | TTATTGTACTGAAGAATGCTTGAA |
|  | OsGA2ox7F | CTCCAGGTGCTAACGAAC |
|  | OsGA2ox7R | ATGGACACCCTCGACTTC |
|  | OsGA2ox8F | CGATTCCTTCTTCGTCAAC |
|  | OsGA2ox8R | AAGTAGATCACGCTCCTG |
|  | OsGA2ox9F | CATCGTCAACATCGGAGA |
|  | OsGA2ox9R | GGATTTGTGTTGAGATGTTTATAC |
|  | OsGA2ox10F | CTCTTTGCGTGATGGTAG |
|  | OsGA2ox10R | TTCGTTAGAACCTGTAAGAC |
|  | OsRPH-F | GTCTCCTCCCTCCTCCTCTC |
|  | OsRPH-R | TAGTAGACGCTGGAGCCGAG |
| Subcellular Localization | GATEWAY-OsRPH-F | CAAAAAAGCAGGCTTCATGGACGCCAGCCTCCGCAC |
|  | GATEWAY-OsRPH-R | CAAGAAAGCTGGGTCCTAGAAGTTAAGCATCTCCC |
| Transactivation Activity Assay | PBR-OsRPH-F | TGTATCGCCGGAATTCATGGACGCCAGCCTCCGCAC |
|  | PBR-OsRPH-R | TTGGCTGCAGGTCGACCTAGAAGTTAAGCATCTCCC |
| Yeast Two-hybrid Assay | AD-CRY1b-F | GGAGGCCAGTGAATTCATGTCGGTGTCGTCGTCGTC |
|  | AD-CRY1b-R | CGAGCTCGATGGATCCCTAACCGATCCAATTTGGTT |
| BiFC | BIFC-CRY1b-103F | AGGTACCCGGGGATCCATGTCGGTGTCGTCGTCGTC |
|  | BIFC-CRY1b-103R | CGCCGTCGACTCTAGACTAACCGATCCAATTTGGTT |
|  | BIFC-CRY1b-105F | CAAGGCCGGCGGATCCATGTCGGTGTCGTCGTCGTC |
|  | BIFC-CRY1b-105R | GCAGGTCGACTCTAGACTAACCGATCCAATTTGGTT |
|  | BIFC-OsRPH-103F | AGGTACCCGGGGATCCATGGACGCCAGCCTCCGCAC |
|  | BIFC-OsRPH-103R | CGCCGTCGACTCTAGACTAGAAGTTAAGCATCTCCC |
|  | BIFC-OsRPH-105F | CAAGGCCGGCGGATCCATGGACGCCAGCCTCCGCAC |
|  | BIFC-OsRPH-105R | GCAGGTCGACTCTAGACTAGAAGTTAAGCATCTCCC |
