# Supplementary material for: A Novel AP2/ERF Transcription Factor, OsRPH1, Negatively Regulates Plant Height in Rice
Source: Front Plant Sci. 2020 May 27;11:709. doi: 10.3389/fpls.2020.00709 (PMC7266880; doi:10.3389/fpls.2020.00709)
Supplement: TABLE S2 — Potential proteins interacted with OsRPH1. [file Table_2.DOCX]

**Supplementary TABLE 2 | Potential proteins interact with OsRPH1**

| Number | Locus Name | Annotation |
| --- | --- | --- |
| 1 | LOC_Os05g49700 | OsCRY1b |
| 2 | LOC_Os04g42095 | S-adenosyl-l-methionine decarboxylase leader peptide, putative, expressed |
| 3 | LOC_Os03g02850 | tobamovirus multiplication protein, putative, expressed |
